# Oncological outcomes of dose reductions in cisplatin due to renal dysfunction for patients with metastatic urothelial carcinoma

**DOI:** 10.1002/bco2.81

**Published:** 2021-03-09

**Authors:** Tetsushi Murakami, Eiji Kikuchi, Hiroki Ide, Yuta Umezawa, Takayuki Takahashi, Mizuki Izawa, Kyohei Hakozaki, Keisuke Shigeta, Koichiro Ogihara, Hiroaki Kobayashi, Kunimitsu Kanai, Takahiro Maeda, Shunsuke Yoshimine, Ryuichi Mizuno, Koshiro Nishimoto, Mototsugu Oya

**Affiliations:** ^1^ Department of Urology Keio University School of Medicine Tokyo Japan; ^2^ Department of Urology Saitama City Hospital Saitama Japan; ^3^ Department of Urology St. Marianna University School of Medicine Kanagawa Japan; ^4^ Department of Urology Tokyo Saiseikai Central Hospital Tokyo Japan; ^5^ Department of UroOncology Saitama Medical University International Medical Center Saitama Japan; ^6^ Department of Urology Kawasaki Municipal Hospital Kanagawa Japan; ^7^ Department of Urology National Hospital Organization Saitama Hospital Saitama Japan; ^8^ Department of Urology Saiseikai Yokohamashi Tobu Hospital Kanagawa Japan

**Keywords:** cisplatin‐unfit, GC, metastatic urothelial carcinoma, renal failure, salvage chemotherapy

## Abstract

**Objective:**

To investigate whether dose reductions in cisplatin due to renal dysfunction were associated with worse clinical outcomes in metastatic urothelial carcinoma (UC) patients.

**Patients and methods:**

One hundred and fifty one metastatic UC patients who received first‐line gemcitabine plus cisplatin (GC) salvage chemotherapy without a previous history of peri‐surgical chemotherapy were included in this retrospective study. Patients with endogenous creatinine clearance of 60 mL/min or more were treated with a full dose of cisplatin, while those with 45‐59 and 30‐44 mL/min were treated with 75% and 50% doses, respectively. Patients were divided into three groups based on the average administered dose of cisplatin of 100% (Group A, N = 43), 99%‐75% (Group B, N = 59), and less than 75% (Group C, N = 49), and therapeutic responses and the toxicity of GC were compared.

**Results:**

Complete response rates were 9.3%, 13.6%, and 14.3% in groups A, B, and C, respectively. One‐year progression‐free survival rates were 22.9%, 31.1%, and 36.7% in groups A, B, and C with no significant differences. One‐year cancer‐specific survival rates were 56.1%, 71.1%, and 68.3% in groups A, B, and C with no significant differences. A multivariate Cox's regression analysis showed that the dose of cisplatin was not an independent prognostic factor for disease progression and cancer death. Furthermore, there were no significant differences in the incidence of severe adverse events.

**Conclusions:**

Dose reductions in cisplatin due to renal dysfunction did not worsen clinical outcomes for metastatic UC.

**Abbreviations**
AUC: area under the curve
CDDP: cisplatin
CR: complete response
CrCl: creatinine clearance
CSS: cancer‐specific survival
GC: gemcitabine plus cisplatin
GCarbo: gemcitabine plus carboplatin
G‐CSF: granulocyte colony‐stimulating factor
GFR: glomerular filtration rate
GP: gemcitabine plus paclitaxel
MVAC: methotrexate, vinblastine, doxorubicin, and cisplatin
PCG: paclitaxel, cisplatin, and gemcitabine
PFS: progression‐free survival
PR: partial response
RNU: radical nephroureterectomy
UC: urothelial carcinoma
UTUC: upper tract urothelial carcinoma

## 1 INTRODUCTION

The gemcitabine plus cisplatin (GC) regimen and high‐dose intensity methotrexate, vinblastine, doxorubicin, and cisplatin (MVAC) regimen with growth factor support are used as first‐line systemic therapies for locally advanced or metastatic urothelial carcinoma (UC). Unfortunately, UC patients with renal impairment (glomerular filtration rate: GFR < 60 mL/min) are classified as cisplatin‐unfit in the current guidelines and account for approximately half of UC patients.^1^, ^2^, ^3^, ^4^, ^5^ Specifically, a total of 37% of patients treated with radical nephroureterectomy (RNU) for upper tract urothelial carcinoma (UTUC) had a preoperative estimated GFR of 60 mL/min/1.73 m^2^ or more, which decreased to 16% after RNU.^5^ Although the gemcitabine plus carboplatin (GCarbo) regimen and immune‐checkpoint inhibitors have been used as first‐line systemic therapies for cisplatin‐unfit UC patients,^6^, ^7^, ^8^ a meta‐analysis of randomized phase II and III trials revealed that cisplatin‐based chemotherapy achieved a significantly higher complete response (CR) and overall response rates than carboplatin‐based therapy in metastatic UC patients with normal renal function.^9^ When the metastatic UC patients with renal impairment become resistant to Anti‐PD‐1/PD‐L1 antibody, those patients receive less effective regimen such as GCarbo, gemcitabine plus paclitaxel (GP) or gemcitabine monotherapy.

Currently, societies of nephrology recommend dose reductions in cisplatin for patients with renal insufficiency of creatinine clearance (CrCl) between 30 and 60 mL/min.^10^, ^11^, ^12^ Among their recommendations, unified protocol based on Kintzel's indication is especially popular.^10^, ^11^, ^13^ Kintzel et al reported an indicator of cisplatin dose reductions in patients with impaired renal function based on empiric adjustment due to nephrotoxicity. This indicator shows that patients with CrCl of more than 60 mL/min received a full dose of cisplatin, while those with 46‐60 and 30‐45 mL/min received 75% and 50% doses, respectively.^13^ However, UC patients with renal impairment (GFR < 60 mL/min) are classified as cisplatin‐unfit in the current urology guidelines.^1^, ^2^, ^3^ The reason why they have different opinions about dose reductions in cisplatin for patients with renal insufficiency of CrCl between 30 and 60 mL/min is that there have been no analyses on the clinical outcomes of reduced cisplatin in metastatic UC patients. Thus, is it appropriate to suggest that all UC patients with renal impairment are cisplatin‐unfit? Dose reductions in cisplatin due to renal dysfunction worsen oncological outcomes in metastatic UC? To elucidate this issue, we investigated whether reduced cisplatin doses in the GC regimen based on endogenous CrCl levels could influence oncological outcomes and associated toxicity than the full dose in patients with metastatic UC.

## 2 PATIENTS AND METHODS

### 2.1 Patient samples

**FIGURE 1:**
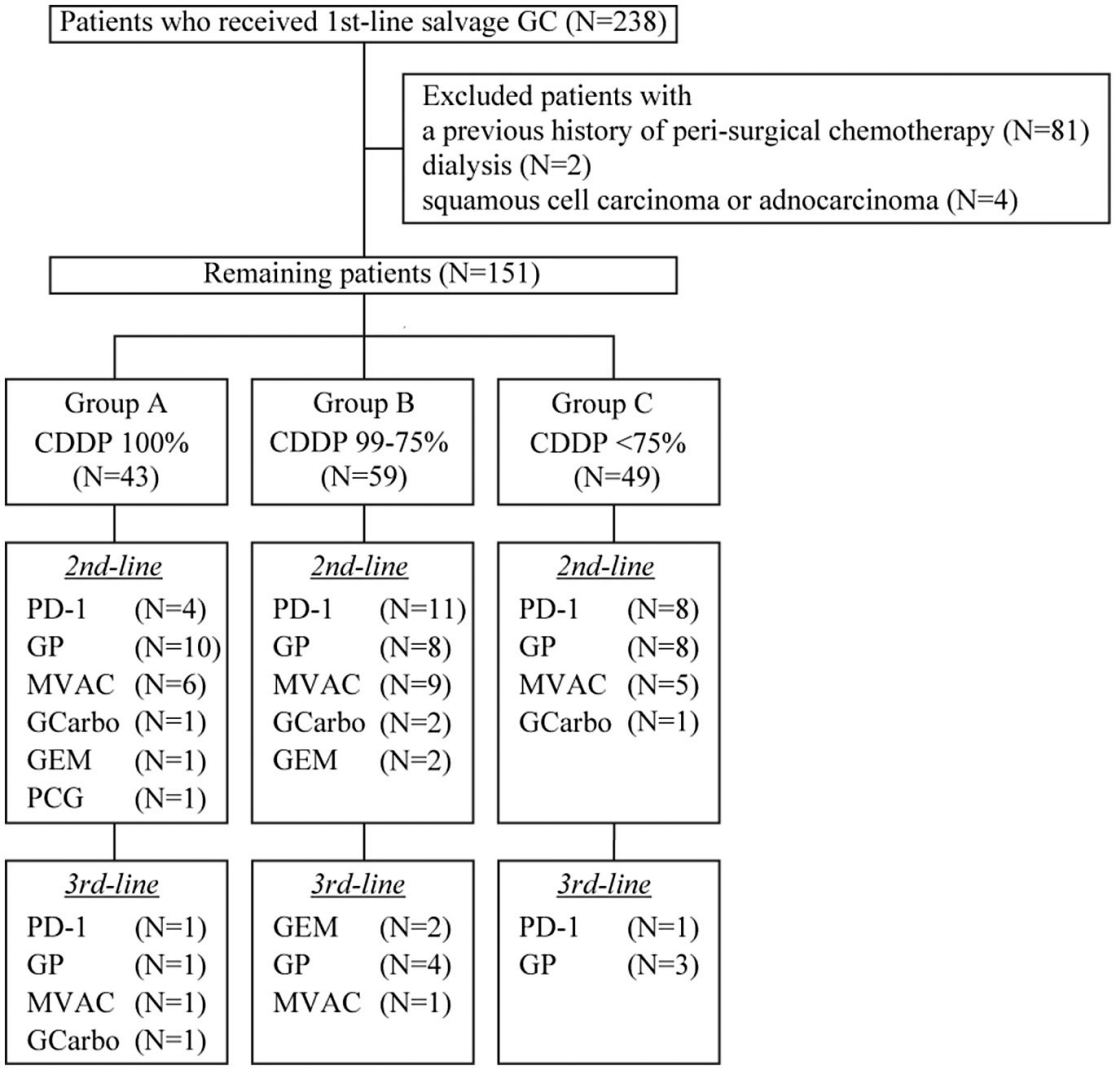
Flow diagram of the study population. GC: gemcitabine plus cisplatin, CDDP: cisplatin, PD‐1: Anti‐PD‐1 antibody, GP: gemcitabine plus paclitaxel, MVAC: methotrexate, vinblastine, doxorubicin, and cisplatin, GCarbo: gemcitabine plus carboplatin, GEM: gemcitabine monotherapy, PCG: paclitaxel, cisplatin, and gemcitabine

We retrospectively identified 238 metastatic UC patients (TanyN1‐3M0 or TanyNanyM1) who underwent first‐line GC salvage chemotherapy between 2008 and 2020 at our seven institutions, consisting of Keio University Hospital and six affiliated institutions. All cases were histopathologically diagnosed with UC by excision or biopsy of the primary lesion or biopsy of a metastatic lesion. Eighty‐one patients with a previous history of peri‐surgical chemotherapy, two patients on dialysis and four patients with evidence of a squamous cell carcinoma or adenocarcinoma histology were excluded from the present study (Figure 1). We divided 151 patients into three groups based on the average administered dose of cisplatin of 100% (Group A, N = 43), 99%‐75% (Group B, N = 59), and less than 75% (Group C, N = 49). We examined clinical backgrounds and therapeutic responses to GC chemotherapy in the three groups.

### 2.2 GC regimen

The GC regimen used in the present study was as follows. UC patients with normal renal function were administered gemcitabine 1,000 mg/m^2^ on days 1, 8, and 15 and cisplatin 70 mg/m^2^ on day 2 every 28 days. Concurrent radiation and split doses of cisplatin were not performed. To select the dose of cisplatin based on Kintzel's indication,^13^ we measured endogenous CrCl using 24‐hour urine specimens obtained immediately prior to each cycle of GC chemotherapy.

According to Kintzel's indication with modifications, patients with endogenous CrCl of equal to or more than 60 mL/min were treated with a full dose of cisplatin, whereas those with endogenous CrCl of 45‐59 and 30‐44 mL/min were treated with 75% and 50% doses, respectively. In contrast, the dose of gemcitabine was not affected by the level of endogenous CrCl. The gemcitabine dose was reduced to approximately 80% according to the physician's discretion as appropriate when repeated grade 3 or 4 adverse events were observed and general status was expected to deteriorate due to adverse effects. Granulocyte colony‐stimulating factor (G‐CSF) was administered when the neutrophil count was less than 500/mm^3^ and was not used prophylactically. The GC dose in each case was calculated based on the average value of all cycles in individuals.

### 2.3 Response evaluation

Tumor measurements were assessed radiologically using computed tomography scans before the start of the GC regimen. All patients were evaluated for their response to treatment after the completion of several cycles. Tumor responses were assessed based on the Response Evaluation Criteria in Solid Tumors (RECIST) version 1.1. CR was defined as the complete disappearance of all evidence of cancer for at least 4 weeks. Partial response (PR) was defined as a more than 30% reduction in the sum of the longest diameters of the target lesions without any new lesions. Stable disease was defined as a less than 30% reduction in the sum of the longest diameters of the target lesions or a less than 20% increase in the size of the lesions without any new lesions. Progressive disease was defined as equal to or more than a 20% increase in the sum of measurable lesions or the appearance of new lesions. Toxicity was graded according to the Common Terminology Criteria for Adverse Events (CTCAE) version 5.0. Progression‐free survival (PFS) was measured from the first day of the GC regimen to the day of the first evidence of disease progression. Cancer‐specific survival (CSS) was calculated from the first day of the GC regimen until death or the last follow‐up.

### 2.4 Statistical analysis

Radical surgery included total cystectomy for bladder cancer and RNU for UTUC. Local recurrence was defined as relapse within the retroperitoneal field of exenteration. Visceral metastasis was categorized as the liver, lung, bone (including bone marrow), and others (including the adrenal gland, peritoneum, skin, and other organs not classified elsewhere). Lymph node metastasis was defined as regional or distant lymphadenopathy.

CSS and PFS curves were estimated by the Kaplan‐Meier method and compared using the Log‐rank test. Clinical and pathological parameters were assessed in multivariate models using Cox's proportional hazard regression models with a stepwise forward selection method. Comparisons of the distribution of binary and non‐ordered categorical variables were performed using the *χ* ^2^ test. Differences in the mean endogenous CrCl values were analyzed using the Mann‐Whitney *U* test. A *P‐*value of less than .05 was considered to indicate significance. Analyses were performed using the SPSS version 26.0 statistical software package.

## 3 RESULTS

### 3.1 Clinical features according to the cisplatin (CDDP) dose administered

The clinical features of patients are shown in Table 1. The median age of 151 patients was 70 years (range, 38‐88 years). The median and average follow‐up period was 12.9 months (range, 1.3‐100.5 months) and 18.1 months, respectively. Information regarding subsequent systemic therapy is shown in Figure 1. Among 151 patients, 43 (28.5%, group A) received a full dose of CDDP, and 108 (71.5%) received a reduced dose as follows: 99%‐75% dose of CDDP group (N = 59, 39.1%, group B) and less than 75% of CDDP group (N = 49, 32.4%, group C). A significant difference was observed in the primary site between groups A and C (*P* = .010) and the incidence of lymph node metastasis between groups A and B (*P* = .016).

**TABLE 1:** Clinical features according to the CDDP dose administered

Characteristics		A	B	C	*P* value		
		CDDP 100%	CDDP 99%‐75%	CDDP < 75%	A vs B	A vs C	B vs C
		n = 43 (%)	n = 59 (%)	n = 49 (%)			
Age	>65	29 (67.4)	42 (71.2)	41 (83.7)	.685	.069	.126
	≤65	14 (32.6)	17 (28.8)	8 (16.3)			
Primary site	UUT	15 (34.9)	27 (45.8)	27 (55.1)	.216	.010	.251
	Bladder	28 (65.1)	30 (50.8)	18 (36.7)			
	Both	0	2 (3.4)	4 (8.2)			
Sex	Male	28 (65.1)	41 (69.5)	31 (63.3)	.641	.853	.494
	Female	15 (34.9)	18 (30.5)	18 (36.7)			
PS	0‐1	43 (100)	59 (100)	46 (93.9)	–	.099	.054
	2	0	0	3 (6.1)			
History of radical surgery	Yes	15 (34.9)	26 (44.1)	25 (51.0)	.350	.119	.471
	No	28 (65.1)	33 (55.9)	24 (49.0)			
Pure UC	Yes	41(95.3)	58(98.3)	49(100)	.382	.216	.546
	No	2(4.7)	1(1.7)	0			
Grade	G1/G2	10(23.3)	11(18.6)	9(18.4)	.599	.671	.939
	G3	33(76.7)	47(79.7)	37(75.5)			
	unknown	0	1(1.7)	3(6.1)			
Lymph node metastasis	Yes	23 (53.5)	45 (76.3)	29 (59.2)	.016	.582	.057
	No	20 (46.5)	14 (23.7)	20 (40.8)			
Visceral metastasis	Yes	27 (62.8)	27 (45.8)	28 (57.1)	.089	.581	.239
	No	16 (37.2)	32 (54.2)	21 (42.9)			
Local recurrence	Yes	6 (14.0)	7 (11.9)	5 (10.2)	.755	.580	.785
	No	37 (86.0)	52 (88.1)	44 (89.8)			

Abbreviations: CDDP, cisplatin; PS, performance status; UC, urothelial carcinoma; UUT, upper urinary tract.

The means ± standard deviations of endogenous CrCl and GC doses among groups of A, B, and C are shown in Table 2.

**TABLE 2:** Mean ± SD of eCrCl and doses of CDDP (%, mg/m^2^) and gemcitabine (%, mg/m^2^) according to the CDDP dose administered

	A	B	C
	CDDP 100%	CDDP 99%‐75%	CDDP < 75%
	n = 43	n = 59	n = 49
eCrCl (mL/min)	87.9 ± 22.5	66.0 ± 18.2	49.7 ± 12.2
CDDP dose (%)	100 ± 0	85.1 ± 7.9	57.3 ± 9.9
CDDP dose (mg/m^2^)	70 ± 0	59.6 ± 5.5	40.1 ± 6.9
Gemcitabine dose (%)	99.2 ± 3.6	97.9 ± 5.11	96.2 ± 8.0
Gemcitabine dose (mg/m^2^)	992.2 ± 35.8	979.3 ± 51.1	961.6 ± 79.7

Abbreviations: CDDP, cisplatin; eCrCl, endogenous creatinine clearance; SD, standard deviation.

### 3.2 Relationship between the CDDP dose administered and oncological outcomes

CR rates were 9.3%, 13.6%, and 14.3%, and PR rates were 18.6%, 40.7%, and 40.8% in groups A, B, and C, respectively. Taken together, clinical response (CR + PR) rates among groups A, B, and C were 27.9%, 54.3%, and 55.1%, respectively (Table 3). The median PFS and CSS time were 4.2, 6.6, and 8.5 months and 9.2, 13.2, and 15.4 months in groups A, B, and C, respectively.

**TABLE 3:** Clinical responses according to the CDDP dose administered

Responses	A	B	C
	CDDP 100%	CDDP 99%‐75%	CDDP < 75%
	n = 43 (%)	n = 59 (%)	n = 49 (%)
CR	4 (9.3)	8 (13.6)	7 (14.3)
PR	8 (18.6)	24 (40.7)	20 (40.8)
SD	9 (20.9)	12 (20.3)	9 (18.4)
PD	22 (51.2)	15 (25.4)	13 (26.5)

Abbreviations: CDDP, cisplatin; CR, complete response; PD, progressive disease; PR, partial response; SD, stable disease.

**FIGURE 2:**
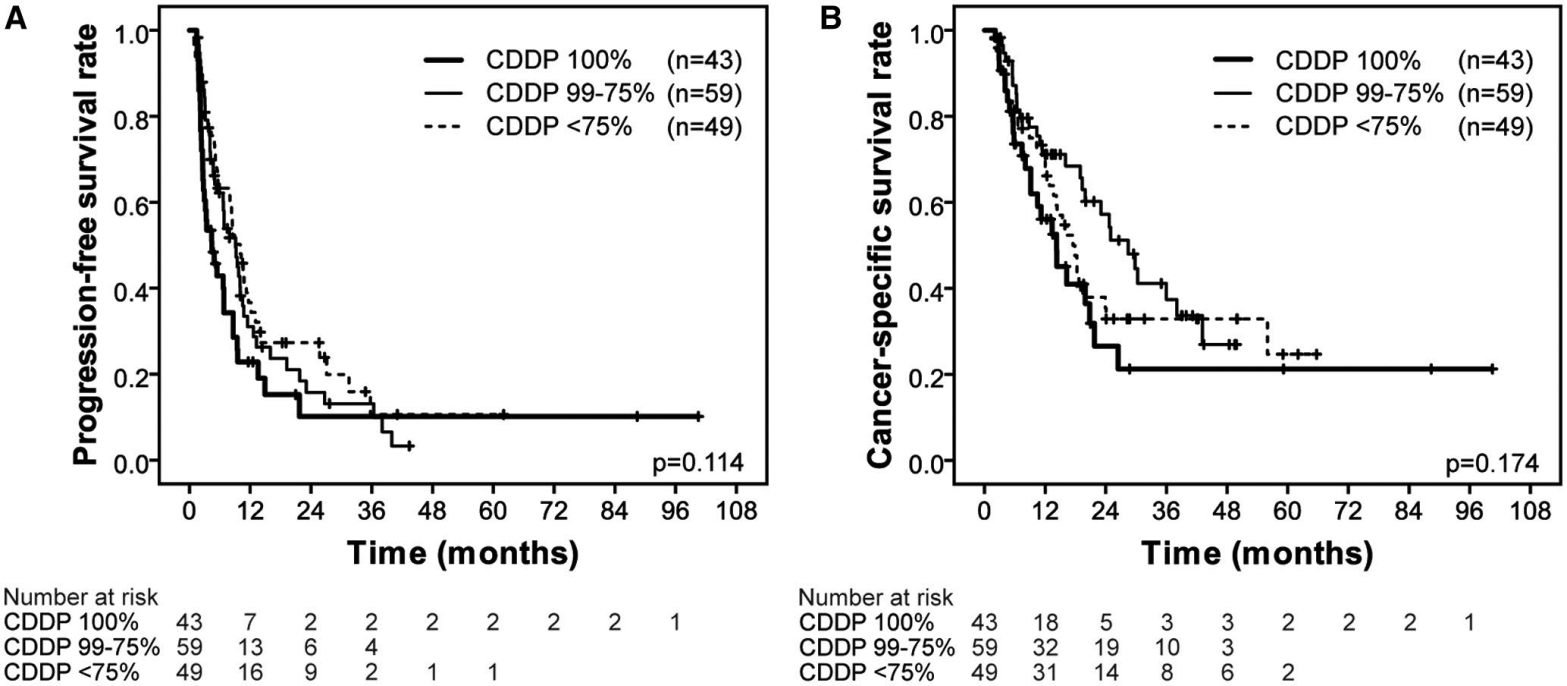
(A) Progression‐free survival of 151 metastatic urothelial carcinoma patients treated with gemcitabine plus cisplatin stratified by the dose of cisplatin (CDDP) administered (CDDP of 100% vs 99%‐75% vs < 75%). (B) Cancer‐specific survival of 151 metastatic urothelial carcinoma patients treated with gemcitabine plus cisplatin stratified by the dose of cisplatin (CDDP) administered (CDDP of 100% vs 99%‐75% vs < 75%)

Cox's univariate analysis identified performance status (*P* = .049) and a history of radical surgery (*P* = .023) as indicators of disease progression (Table 4). A multivariate Cox's regression analysis showed that history of radical surgery (hazard ratio, 0.621; *P* = .015) was independently associated with disease progression. The initial renal function based on the endogenous CrCl at the first cycle of GC chemotherapy (endogenous CrCl ≥ 60 mL/min vs endogenous CrCl < 60 mL/min) and the CDDP dose administered (CDDP ≥ 75% vs CDDP < 75%) were not associated with disease progression. The Kaplan‐Meier curve showed 1‐year PFS rates of 22.9%, 31.1%, and 36.7% in groups A, B, and C, respectively, which were not significantly different (*P* = .114; Figure 2a).

**TABLE 4:** Uni‐ and multivariate Cox's regression analyses of disease progression and cancer‐specific death

Clinical indicators	Disease progression						Cancer‐specific death					
	Univariate			Multivariate			Univariate			Multivariate		
	HR	95% CI	*P* value	HR	95% CI	*P* value	HR	95% CI	*P* value	HR	95% CI	*P* value
CDDP ≥ 75% vs CDDP < 75%	1.399	0.963‐2.032	.078				0.917	0.597‐1.411	.695			
eCrCl ≥ 60 mL/min vs < 60 mL/min	1.235	0.828‐1.844	.301				1.025	0.645‐1.629	.916			
Age > 65 vs ≤ 65	0.671	0.449‐1.001	.050				0.516	0.323‐0.824	.006	0.497	0.310‐0.796	.004
Sex (male vs female)	0.836	0.569‐1.229	.362				0.689	0.444‐1.069	.097			
PS (2 vs 0‐1)	3.214	1.004‐10.292	.049				1.524	0.374‐6.216	.557			
History of radical surgery (Yes vs No)	0.648	0.446‐0.942	.023	0.621	0.423‐0.912	.015	0.630	0.406‐0.979	.040			
Primary site (BT vs UTUC)	1.048	0.774‐1.418	.762				1.158	0.806‐1.665	.427			
Lymph node metastasis (Yes vs No)	0.882	0.606‐1.283	.510				0.817	0.530‐1.260	.360			
Visceral metastasis (Yes vs No)	1.207	0.837‐1.739	.314				1.511	0.975‐2.343	.065			
Local recurrence (Yes vs No)	1.467	0.885‐2.431	.137				1.176	0.638‐2.167	.604			

Abbreviations: BT, bladder tumor; CDDP, cisplatin; CI, confidence interval; eCrCl, endogenous creatinine clearance; HR, hazard ratio; PS, performance status; UTUC, upper tract urothelial carcinoma.

Cox's univariate analysis identified age (*P* = .006) and a history of radical surgery (*P* = .040) as indicators of cancer‐specific death (Table 4). The multivariate Cox's regression analysis showed that age (hazard ratio, 0.497; *P* = .004) was an independent prognostic factor for cancer death. The initial renal function and the CDDP dose administered were not identified as predictors of cancer death. The Kaplan‐Meier curve showed 1‐year CSS rates of 56.1, 71.1, and 68.3% in groups A, B, and C, respectively, which were not significantly different (*P* = .174; Figure 2b).

### 3.3 Adverse events according to the CDDP dose administered

No significant differences were observed in overall toxicity between the three groups. Grade 3/4 leukopenia occurred in 30.2%, 37.3%, and 36.7% of patients in groups A, B, and C, respectively (Table 5). Grade 3/4 neutropenia occurred in 41.8%, 52.5%, and 57.1% of patients in groups A, B, and C, respectively. Respective percentages for grade 3/4 thrombocytopenia were 58.1%, 49.1%, and 53.1%. Three out of 151 patients had grade 3 acute kidney injury (1 in group A and 2 in group C).

**TABLE 5:** Adverse effect profile according to the CDDP dose administered

Toxicity (Grade)	A			B			C		
	CDDP 100%			CDDP 99%‐75%			CDDP < 75%		
	Number (%)			Number (%)			Number (%)		
	Grade 3	Grade 4	Grade 3/4	Grade 3	Grade 4	Grade 3/4	Grade 3	Grade 4	Grade 3/4
Leukopenia	12 (27.9)	1 (2.3)	13 (30.2)	20 (33.9)	2 (3.4)	22 (37.3)	15 (30.6)	3 (6.1)	18 (36.7)
Neutropenia	9 (20.9)	9 (20.9)	18 (41.8)	21 (35.6)	10 (16.9)	31 (52.5)	18 (36.7)	10 (20.4)	28 (57.1)
Thrombocytopenia	19 (44.2)	6 (13.9)	25 (58.1)	17 (28.8)	12 (20.3)	29 (49.1)	16 (32.7)	10 (20.4)	26 (53.1)
Febrile neutropenia	2 (4.7)	0	2 (4.7)	0	0	0	3 (6.1)	0	3 (6.1)
Acute kidney injury	1 (2.3)	0	1 (2.3)	0	0	0	2 (4.1)	0	2 (4.1)
Gastric hemorrhage	0	0	0	1 (1.7)	0	1 (1.7)	1 (2.0)	0	1 (2.0)
Vertigo	0	0	0	1 (1.7)	0	1 (1.7)	1 (2.0)	0	1 (2.0)
Constipation	0	0	0	1 (1.7)	0	1 (1.7)	0	0	0

Abbreviation: CDDP, cisplatin.

Among 151 patients, the trends of endogenous CrCl values immediately prior to the first, third, and fifth cycles for each group are shown in Figure S1. In group A, the mean endogenous CrCl value obtained just before the first cycle of chemotherapy was 95.3 mL/min, which was not significantly higher than that immediately prior to the third (84.1 mL/min, *P* = .129) and fifth cycles (80.7 mL/min, *P* = .099). In group B, the mean endogenous CrCl value obtained just before the first cycle of chemotherapy was 83.4 mL/min, which was significantly higher than that immediately prior to the third (64.3 mL/min, *P* = .024) but not different from that immediately prior to the fifth cycles (65.3 mL/min, *P* = .252). In group C, the mean endogenous CrCl value obtained just before the first cycle of chemotherapy was 54.9 mL/min, which was not significantly higher than that immediately prior to the third (51.8 mL/min, *P* = .517) and fifth cycles (48.1 mL/min, *P* = .209).

## 4 DISCUSSION

In the present study, we investigated the oncological outcomes of dose reductions in cisplatin due to renal dysfunction for patients with metastatic UC. To better assess the clinical response to reduced cisplatin, we focused only on metastatic UC cases that received first‐line salvage GC. Because their renal function with each cycle often changes and there are no helpful previous studies, we calculated the dosage of cisplatin adjusted according to endogenous CrCl in each case based on the average value of all cycles in individuals. By the use of the average dosage, we divided advanced UC patients who received GC into the following three groups: cisplatin equal to a 100% dose vs equal to or more than a 75% but less than 100% dose vs less than a 75% dose and revealed two major findings.

First, our metastatic UC cohort showed that the reduced dose of cisplatin did not have a negative impact on the cancer‐specific prognosis of metastatic UC patients with renal dysfunction of endogenous CrCl of 30‐60 mL/min. Moreover, no significant differences were observed in grade 3/4 leukopenia, neutropenia, thrombocytopenia, febrile neutropenia, and acute kidney injury between the full and reduced dose groups. Our results support the recommendation of renal associations regarding the reduced cisplatin.^10^, ^11^, ^12^, ^13^ This means that gemcitabine plus a reduced dose of cisplatin has potential as a therapeutic option for metastatic UC with endogenous CrCl of 30‐60 mL/min. In fact, nationwide survey of 627 institutes in Japan revealed that 54.9% of the institutes use gemcitabine plus a reduced dose of cisplatin and 27.1% of the institutes use the GCarbo regimen for metastatic UTUC patients with impaired renal function (GFR of 30‐59 mL/min).^14^

According to our results, the reason why patients in group A had a higher incidence of PD as compared to those in groups B and C has not been fully explained. However, the difference in clinical backgrounds between the groups might have had an influence on the clinical responses to GC therapy. In fact, the presence of visceral metastasis such as liver metastasis was higher in group A as compared to groups B and C.

Second, the initial renal function (endogenous CrCl < 60 mL/min) at the first cycle of GC chemotherapy was not an independently poor prognostic factor for metastatic UC patients treated with gemcitabine plus a reduced dose of cisplatin. These results showed that patients with renal insufficiency of endogenous CrCl between 30 and 60 mL/min do not have poor oncologic outcomes under the optimal dose reductions in cisplatin.

To the best of our knowledge, only one study reported the effects of reduced dose and standard dose of GC for the metastatic UC patients with renal impairment (GFR < 60 mL/min/1.73 m^2^)^15^; the 1‐year overall survival rate in 25 metastatic UC patients who received a reduced dose was 26.3%, which was significantly lower than that in 32 patients who received the standard dose (60.3%). However, the dose reduction protocol for the GC regimen was not clarified in this study. Moreover, two studies reported the outcomes of metastatic UC patients with renal impairment. Maru et al reported the influence of baseline renal function and dose reduction of nephrotoxic chemotherapeutic agents on the outcome of metastatic UC; a median overall survival in 22 metastatic UC patients who received reduced dose was 10 months, which was significantly lower than that in 35 patients who received the standard dose (median of 17 months). This retrospective study consists of different types of regimens which include only three cases of MVAC and GC in the reduced dose group.^16^ Hsieh et al reported that cisplatin‐fit renal function (CrCl > 60 mL/min) was an independently good prognostic factor for metastatic UTUC patients treated with cisplatin‐based chemotherapy (MVAC; N = 63, GC; N = 73).^17^ The dose reduction protocol for the cisplatin‐based regimen was not clarified in this study either. Compared with previous reports, our cohort study was organized by a single GC regimen under unified protocol of reduced cisplatin with more metastatic UC patients. Furthermore, we consistently measure endogenous CrCl based on 24‐hour urine specimens without using the GFR formula. In our previous study that investigated the relationship between endogenous CrCl and renal function values obtained using mathematical formulas (the Cockcroft‐Gault, Modification of Diet in Renal Disease, Chronic Kidney Disease Epidemiology Collaboration, and Wright formulas), all four formulas appeared to underestimate endogenous CrCl.^18^

Cisplatin binds to blood plasma proteins, binding exceeds 90% within a few hours of its administration, and only free (non‐binding) cisplatin is cytotoxic.^19^, ^20^, ^21^ The plasma level of free cisplatin is important for anti‐tumor activity and nephrotoxicity.^22^, ^23^, ^24^ Since the area under the curve (AUC) of free cisplatin is higher and total body clearance is lower in patients with chronic renal failure than in those with normal renal function, dose reductions in cisplatin are considered for these patients. Gemcitabine is rapidly metabolized to difluorodeoxyuridine by cytidine deaminase and disappears from plasma. The peak plasma concentration, elimination half‐life, clearance, and AUC of gemcitabine are not changed regardless of renal function.^25^, ^26^ Gemcitabine is cytotoxic, whereas difluorodeoxyuridine is considered to be a non‐cytotoxic metabolite.

The present study had several limitations. It was performed with a retrospective and non‐randomized design and a small sample size. Furthermore, we limited the patient cohort to first‐line GC salvage chemotherapy only in order to clarify the appropriateness of reduced GC. The inclusion of metastatic UC patients only in our affiliated institutions may have created a selection bias. Response evaluation assessed by several radiologists at each institution may also be a limitation of the present study.

Finally, the present results showed that dose reductions in cisplatin based on renal function did not worsen clinical outcomes in metastatic UC patients. Accordingly, our results expand the definition of cisplatin‐fit and suggest that UC patients might receive reduced doses of cisplatin even if they have renal failure (endogenous CrCl between 30 and 60 mL/min).

## 5 CONCLUSIONS

We demonstrated that gemcitabine plus a reduced dose of cisplatin did not have a negative impact on clinical outcomes and has potential as a therapeutic option for metastatic UC with endogenous CrCl of 30‐60 mL/min.

## CONFLICT OF INTEREST

There are no conflicts of interest.

## Supporting information

Fig S1
