# Supplementary figures and images for: Oncological outcomes of dose reductions in cisplatin due to renal dysfunction for patients with metastatic urothelial carcinoma
Source: BJUI Compass. 2021 Mar 9;2(5):322–30. doi: 10.1002/bco2.81 (PMC8988766; doi:10.1002/bco2.81)

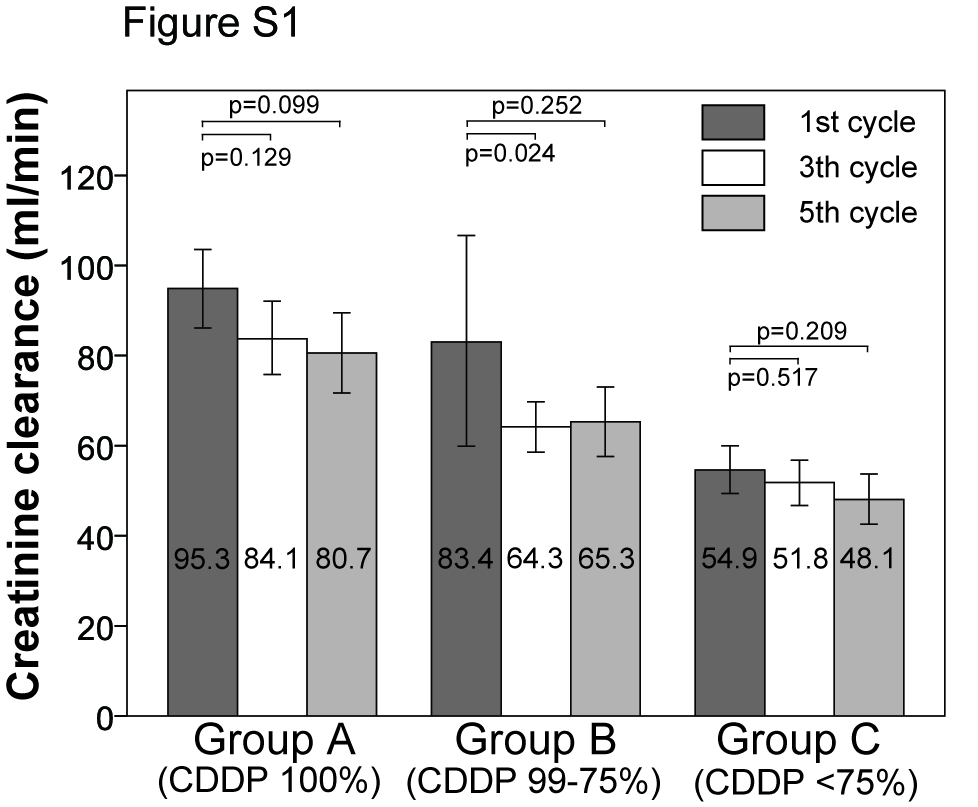

Supplement: Supplementary file 1 — Fig S1 [file BCO2-2-322-s001.tif]
